# Multichannel pulse high-current driver of magnetic actuator

**DOI:** 10.1016/j.ohx.2022.e00286

**Published:** 2022-03-05

**Authors:** Łukasz Bujnowicz, Marcin Sarewicz

**Affiliations:** Department of Molecular Biophysics, Faculty of Biochemistry, Biophysics and Biotechnology, Jagiellonian University, Kraków, Poland

**Keywords:** Stopped flow, Freeze quench, Kinetics of reactions, Piston drive, Supercapacitors

## Abstract

Magnetic linear actuators have a wide range of applications. Their main advantage is the ease with which they can be controlled by regulating the current. However, high electrical power is required for obtaining a large continuous force, acceleration, and stroke from a device with small dimensions. In this study, we developed a comprehensive open-source system consisting of simple movable iron magnetic actuators, a four-channel controller, and dedicated software. The graphical user interface facilitates the designing of the sequence of piston strokes, including the start time, duration of movement, and force for each stroke separately. The controller generates a high current of pulses, which allows achieving a high force, acceleration, and stroke from small-sized coils while maintaining a relatively safe voltage. The system was originally designed as a reagent syringe driver to control the rapid mixing process used for studying the kinetics of enzymatic reactions. However, this driver may also be applied in various other scientific as well as nonscientific applications.

## Graphical abstract

**Figure:**
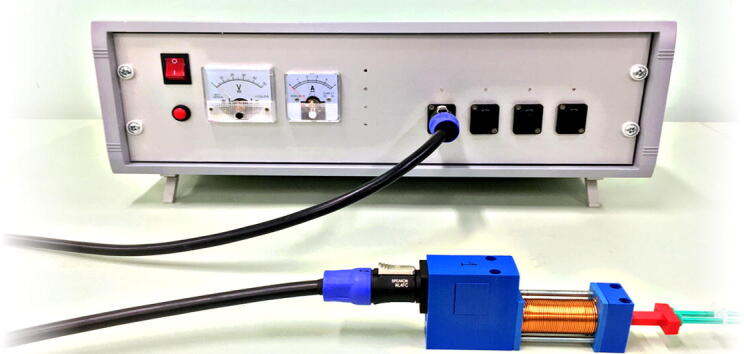

**Specifications table.**

**Table:** 

Hardware Name	Pulse high-current driver of magnetic actuator
Subject Area	• Engineering and Material Science Chemistry and Biochemistry
Hardware Type	• Biological sample handling and preparation Mechanical engineering and materials science
Open Source License	CERN Open Hardware Licence (OHL) - Strongly Reciprocal
Cost of Hardware	1000 EUR
Source File Repository	https://doi.org/10.17632/rvs6gf4sn2.4

## Hardware in Context

Laboratory techniques applied in the studies of chemical and biochemical reactions, such as stopped-flow or freeze-quench, require drivers that can push the plungers of syringes containing the reagents at a considerable speed and force. The driving mechanism should exert a force of tens to hundreds of newtons on the syringe plunger to ensure high flux for proper mixing of the reagents at well-defined time points and for minimizing the dead-time between mixing and reaction monitoring. Various driving device approaches have been investigated so far. The simplest method is to push the pistons manually with hands [1]. Another basic solution is the use of a low-cost spring-actuated piston [2]. More sophisticated pneumatic [3], [4] or hydraulic devices are also commonly used [5]. Recently, actuators based on precise stepper motors have been developed [6]. However, they have some disadvantages which limit their application as versatile open-source projects in laboratory research. The use of a manual driving mechanism makes it difficult to achieve reproducible results in experiments. In the case of spring-actuated mechanism, the piston movement force cannot be effectively adjusted, while pneumatic or hydraulic devices are complicated. Additionally, in spring-actuated, pneumatic, and hydraulic devices, the reverse movements of pistons is mechanically blocked after the shots. Stepper motor-based actuators can help to overcome these problems, but they have their own disadvantages. For some experimental applications, the linear speed of pistons should be more than 1 m/s which can be realized only with the use of specialized and expensive actuators.

In this study, we developed an open-source and universal system based on a magnetic actuator that can be used in experiments requiring controlled movement of the plungers of syringes containing reagents to allow rapid mixing or ejection of the reagents at a significant speed and force. The proposed system enables to control the force applied during the movement as well as the release of the piston, and also provides a high acceleration. Moreover, the system is simple to build and operate, inexpensive, and can be safely used without the risk of fatal electric shock. The system comprises a set of four magnetic coils powered with a stable battery consisting of capacitors that can provide a stable and high current at a relatively safe voltage not exceeding 50 V. This facilitates the pistons to move at a considerable speed and force.

This paper describes in detail the open-source, four-channel, high-current power supply which allows controlling the effective power by pulse-width modulation (PWM). Additionally, we provide binary files and source code of the software dedicated to this device for Linux and Windows systems. Our goal was to use well-known electronic components available to all potential users and necessary instructions to make the assembly process easy for those with only basic electronic knowledge, while the software’s source code allows anyone with a basic understanding of LabVIEW to make any improvements or modifications of the original program.

Although the power supply presented here was originally designed as a part of syringe-driving device, it can be used for any purposes requiring periodic high current with PWM. Due to the availability of four independent channels, it is possible to design more complicated experiments in which more than two reagents can be mixed sequentially, such as stopped-flow or freeze-quench studies.

## Hardware description

The proposed driver of magnetic actuator is a modular structure that can be easily modified.

The electronic system consists of six printed circuit boards (PCBs) listed below:.

**Fig. 1:**
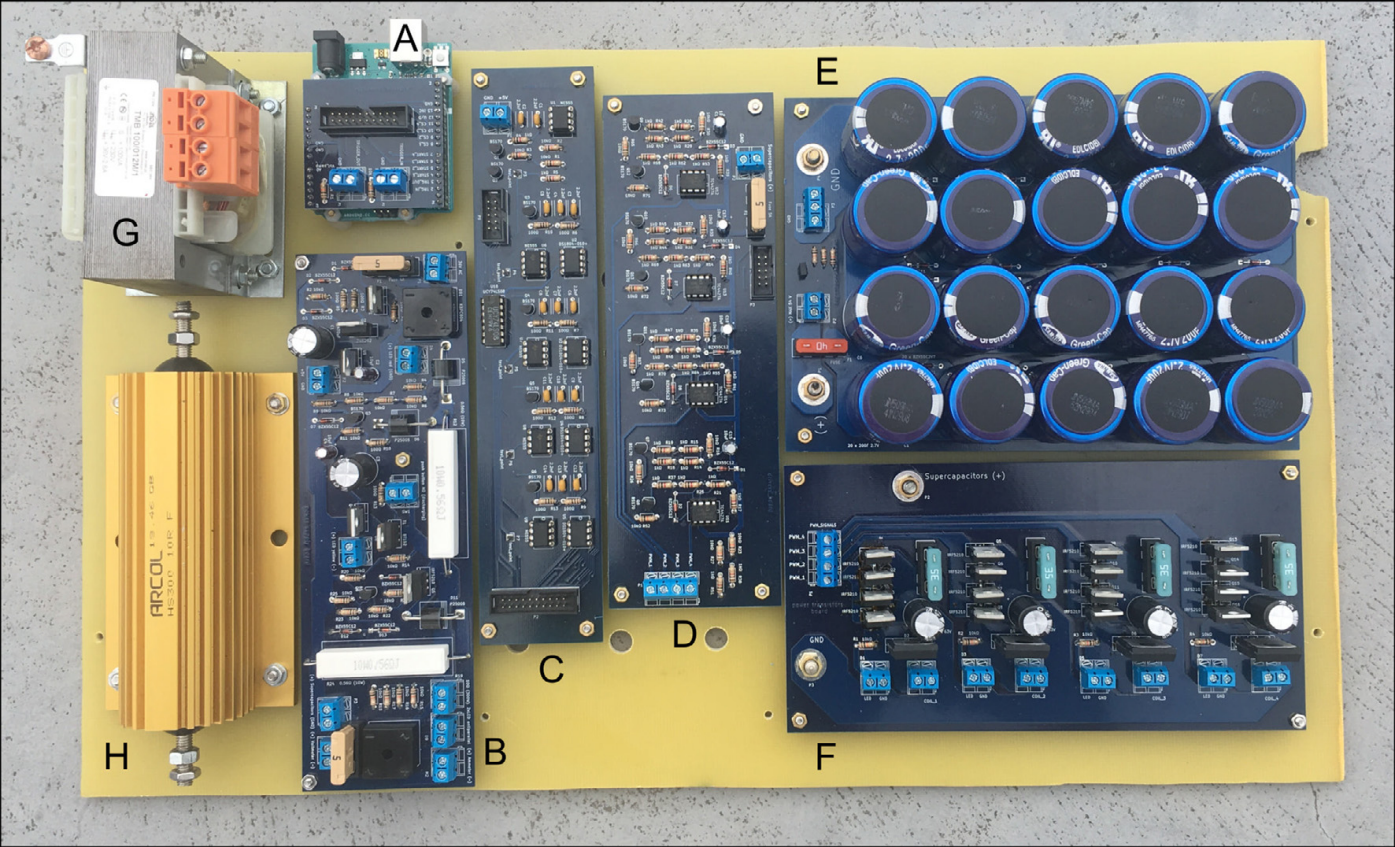
Elements on the mounting board. A) Arduino Uno with Arduino connector board, B) power supply board, C) PWM board, D) drivers board, E) supercapacitors, F) transistors board with the power amplifiers, G) transformer, and H) high-power (300-W) resistor. The figure is available in download files.
• a board controlling the charging/discharging process of supercapacitors (Fig. 1B);
• a board responsible for the formation of the PWM signal (Fig. 1C);
• a board of drivers for power amplifiers (Fig. 1D);
• a board of battery of supercapacitors (Fig. 1E); and
• a board of power amplifiers for four channels (Fig. 1F).

**Fig. 2:**
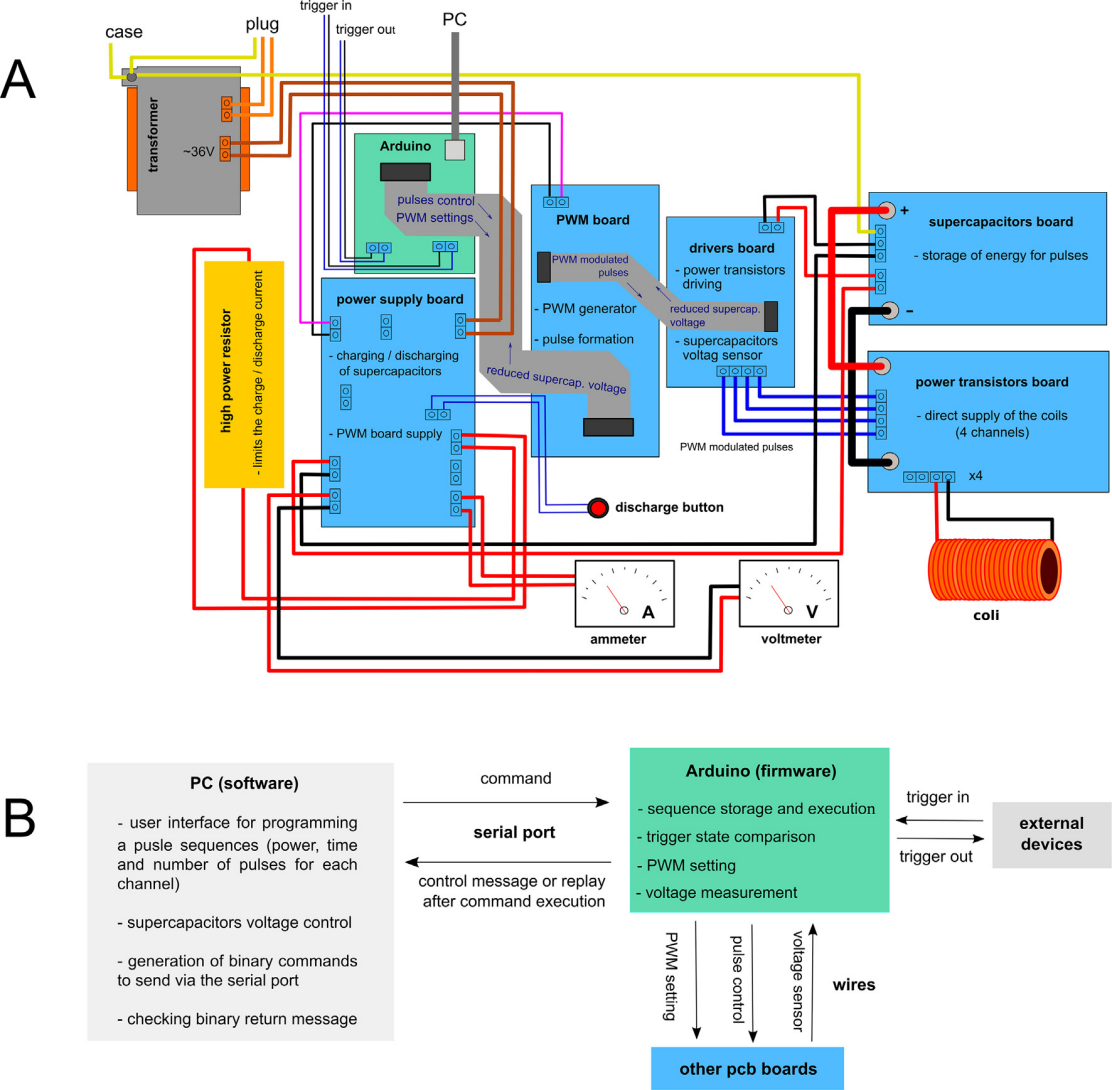
A) Block diagram of the device describing the functions of individual modules/components and illustrating the connections between them. Positive supply cables are shown in red (45 V) and pink (5 V) and negative supply cables in black (their voltage is equal to the ground voltage), all signals are sent through wires shown as navy blue lines except the ribbon cables, USB is shown in gray, and only the signal description is in navy blue. Yellow cables indicate ground. Orange and brown lines show the mains electricity voltage and 36-V cables of AC voltage, respectively. Terminal blocks without output wires correspond to control LEDs, which are not shown for clarity of the drawing. For the same reason, the terminals of only one channel on the power transistors board are presented. B) Block diagram illustrating the interaction between the Arduino microcontroller, the PC software, and the rest of the hardware. The software and firmware functions are listed in the rectangles that represent PC and Arduino microcontroller. Arrows represent information transfer. (For interpretation of the references to color in this figure legend, the reader is referred to the web version of this article.)

• an Arduino Uno controller allowing pulse sequence control and communication with a personal computer (PC) (Fig. 1A);

The device was primarily designed for experiments that require a high power (up to several kW), for not more than a few hundred milliseconds (time of movement of a syringe’s plunger), followed by a few minutes of idle for sample preparation. Such a high power can be possibly applied to the coils directly from the mains electricity, but it can lead to severe electric shock and issues related to overloading of the electrical wiring. Periodic operation of the device can help overcome these problems and limit the accumulation of energy released during piston movement. To prevent the risk of electric shock, the operating voltage should be reduced, which is compensated by an increase in the currents delivered to the coils. This, however, is associated with greater energy losses on system components and imposes a need for wires with larger cross sections. The use of 50 V in the proposed device allows a good compromise between safety and efficiency. While the applied voltage may cause an unpleasant tingling sensation or even pain, it is rather unlikely to be life-threatening to a healthy person under normal working conditions [7]. When using a voltage of ∼ 50 V, the amperage needed to achieve the appropriate power of the device is ∼ 100 A. Considering the short duration of the current pulses, on the order of several hundred milliseconds, this solution allows the use of wires with a sufficiently small diameter to build small coils and connectors. On the other hand, the current is so high that it necessitates dedicated engineering solutions for precise pulse control, energy accumulation, and release. Each of the designed boards performs specific functions essential for the working of the device. Fig. 2A shows a diagram of the device, specifying the functions of individual modules/elements and illustrating the connections between them.

### Supercapacitors board

We used a battery of supercapacitors as an energy source. It allows the accumulation of a large charge while providing high currents during the discharge process. Moreover, the supercapacitors can be quickly charged before the experiment and safely discharged after the experiment. At the same time, they do not pose the risk of uncontrolled discharging after the device is turned off. As the rated voltage of each capacitor is as low as 2.7 V, the capacitors must be connected in series to provide a voltage of around 45–50 V.

### Power supply board

The circuit controlling the charging and discharging processes of the supercapacitors through a high-power resistor was designed on a separate PCB. Supercapacitors should not be charged and discharged rapidly to prevent overloading of the device components. On the other hand, the time required for charging should be minimized so that the operator does not have to wait too long for the device to be ready. Therefore, the designed circuit reduces the charging resistance when the voltage on supercapacitors exceeds 25 V. Furthermore, charging and discharging cannot be triggered simultaneously, as this can lead to a short circuit. Charging/discharging of supercapacitors can be monitored through an analog ammeter and voltmeter fixed in the front panel of the device. Additionally, control diodes on the front panel display the state of the device such as charging or discharging. A power supply for the PWM signal generator is also placed on the board. The entire system is powered by a transformer with an effective output voltage of 36 V and a power of 100 VA.

### Power amplifier board and driver board

Power amplifiers are made of P-MOSFETs (P-metal–oxide–semiconductor field-effect transistors) connected in parallel, which offers a ground for the coils between the pulses, thus preventing accidental short-circuiting. Although P-MOSFETs are relatively difficult to drive than N-MOSFETs, they are significantly safer in this case. Overheating of the power amplifiers can be avoided by using MOSFET drivers that can supply a high current to the gate of the transistors. These drivers are located on a separate board. Although, this split is not an optimal solution, because MOSFET drivers should be located close to the transistors, separate boards enable further modifications of the device. A voltage probe (R23, R27, R38, and R51) is also located on the drivers board.

### PWM board

The controller allows adjusting the effective power delivered to coils by changing the duty cycle of the PWM signal (width of micropulses during the pulse). A PWM signal of constant frequency is generated separately for each channel, regardless of whether the voltage is applied to the coils or not. Only after an additional signal is generated from the Arduino to the AND logic gate, the PWM signal is transmitted to the power amplifier. The generated signal can be previewed on the oscilloscope using dedicated pins on the board. The PWM signal is generated using the NE555 chip, which is easily available and well-documented. The first NE555-based circuit works like a clock with a constant frequency, while the other NE555-based circuits are responsible for the duty cycle regulated by a digital potentiometers. A digital potentiometers facilitate quick adjustment of the duty cycle through software. Although it is old-fashioned, the NE555-based system has one main advantage: it allows the user to modulate the clock frequency and the range of duty cycle only by changing appropriate resistors (R1–R3) and/or capacitors (C3–C14) on the PWM board. The amount of electronic expertise required is minimal because the resistors and capacitors can be easily fitted in a very intuitive and simple online circuit simulator in which the NE555 chip can be found [8]. The PWM signal has a frequency of around 20 kHz, which is so high that the signal is averaged by coils working as low-pass filters. This results in smooth piston movement and stroke, even on a millisecond time scale, while the frequency is low enough to prevent overheating of the power transistors when they are switched on and off.

A PWM board is not absolutely necessary because the Arduino microcontroller can also generate a PWM signal. To avoid using this board, the default Arduino PWM signal frequency of 490 Hz has to be increased to around 30 kHz. This can be done at the firmware level after its reprogramming. Additionally, the ribbon cable should be adapted in such a way that it connects Arduino directly to the drivers board. However, we decided to use a separate PWM board as the project’s goal is to limit dependencies on external solutions as much as possible, while keeping individual functionalities distributed among independent modules, in order to ensure easy device modification. Because PWM modulation is crucial for the working of the device, a separate board was designed to generate the PWM signal.

### Arduino

The actuator driver is controlled by Arduino Uno board which is responsible for communicating with PC, executing pulse sequences, monitoring voltage, and synchronizing with other external components. Arduino is a popular and widely available microcontroller [9]. In this paper, we provide an appropriate source code of the firmware written for Arduino Uno, which is compatible with the included PC software that allows creating our own pulse sequences in the easy graphical user interface. Fig. 2B presents a diagram for communication between the Arduino firmware dedicated to the device and the PC software. The description of the communication protocol is also available for download.

### Coil

In addition to the power supply, the project focused on coils and movable piston cores. The spools of the coil and the handles were prepared as a set for three-dimensional (3D) printing. Our design assumes that a nut will be screwed on a movable core made of M6 bolt. These elements are commercially available.

### Software

The dedicated PC software was created in LabVIEW 2017 and is accessible as source code and binary executable for Linux and Windows. A separate document contains the manual describing all the functions implemented in the program for controlling the device from a PC.

### Application of the device

The described device can be used:.

• as a syringe driver for determining the kinetics of chemical or enzymatic reactions after fast mixing;
• in applications requiring a fast actuator;
• as a pulse source of high local magnetic field (as in the case of a coil-gun);
• as a pulse source of high current.

## Design files

PCBs of electronic parts are available in two formats delivered as two separate packages: EJKL_gerber.zip and EJKL_Kicad.zip. The EJKL_gerber.zip package contains the gerber files prepared for ordering, while the EJKL_Kicad.zip package consists of files that provide complete documentation of the PCBs allowing modification of the device and a dedicated component library of custom footprints and 3D models of elements. The README.txt file included in the EJKL_Kicad.zip package provides instructions for the proper import of the library. The schemes of PCBs are available in the Kicad package and also in a separate EJKL_PCBs_schemes package containing pdf files with schematics.

EJKL_3D_boards.zip package contains png files showing 3D visualization of PCBs. The previews of PCBs can be useful in soldering if one is not familiar with electronic symbols and footprints and also help in soldering the components without installing KiCad software for PCB visualization.

OpenSCAD files in the EJKL_openscad.zip package contain the source code of designed 3D printing and CNC (computer numerical control**)** machining. The files provide information on the following elements: the spool of the coil, the coil holder, the piston, the spacer sleeve for PCBs (3D printing), the front and back panels of the case, and the mounting board for PCBs (CNC machining). EJKL_stl.zip package contains the stl files of these elements ready for manufacturing or 3D printing.

EJKL_firmware.zip package contains a script of firmware for Arduino Uno, which has to be uploaded to the microcontroller to allow communication between the device and PC by a serial port. The same script is included in the dedicated software and can also be saved on a PC directly from the installed PC software. Additionally, the package has a file that precisely describes the protocol of communication between Arduino and PC by a serial port. The description may be useful for developing custom software controlling the device in other languages such as Python.

EJKL_elements.ods package lists all the elements, symbols, prices, and links to e-shops.

Pictures_movie.zip package contains all figures of the manuscript and high resolution image of PCBs on a mounting board and wiring which may be useful for device assembling. It also has a record of tests of boards, assembling, and experimental test described below.

EJKL_Linux64.zip package contains a binary executable file of the software for 64-bit Linux OS. This was compiled with LabVIEW 2017 and requires LabVIEW Runtime Engine 2017 and the latest NI VISA package. The runtime and NI VISA are not included in the package.

EJKL_Win64.zip package contains an installer of binary executable file of the software for 64-bit Windows 7 or newer. This installer provides all libraries needed for running the software and does not necessitate NI VISA or LabVIEW Runtime Engine separately.

EJKL_software_source.zip package contains llb file including source code of all VIs. This file can be loaded to LabVIEW 2017 or newer version, provided that NI VISA has been installed.

EJKL_software_manual.odt is a LibreOffice document containing a manual for the software.

example.ejkl is an example sequence that can be loaded in the software.

All packages and files can be downloaded from the Mendeley repository, the links to which are given in the table below. Additionally, all files, except for the pictures_movie package and the compiled version of the software for Windows, are available at https://github.com/LukaszBujnowiczLab/EJKL_electronics.

## Design files summary

**Table:** 

Design file name	File type	Open source license	Location of the file
EJKL_Kicad.zip	KiCad project	CERN-OHL-S	http://dx.https://doi.org/10.17632/rvs6gf4sn2.4
EJKL_PCBs_schemes.zip	Pdf files with PCB schemes	CERN-OHL-S	http://dx.https://doi.org/10.17632/rvs6gf4sn2.4
EJKL_gerber.zip	Gerber and drill files	CERN-OHL-S	http://dx.https://doi.org/10.17632/rvs6gf4sn2.4
EJKL_3D_boards.zip	PNG images (3D visualization of PCBs)	CERN-OHL-S	http://dx.https://doi.org/10.17632/rvs6gf4sn2.4
EJKL_openscad.zip	.scad projects (3D printing or CNC milling)	CERN-OHL-S	http://dx.https://doi.org/10.17632/rvs6gf4sn2.4
EJKL_stl.zip	.stl files (3D printing or CNC milling)	CERN-OHL-S	http://dx.https://doi.org/10.17632/rvs6gf4sn2.4
EJKL_firmware.zip	Source code for Arduino and text file with communication protocol	CERN-OHL-S	http://dx.https://doi.org/10.17632/rvs6gf4sn2.4
EJKL_elements.ods	List of all elements with links and prices	CERN-OHL-S	http://dx.https://doi.org/10.17632/rvs6gf4sn2.4
pictures_movie.zip	PNG,JPG,MP4 and MPG	CERN-OHL-S	http://dx.https://doi.org/10.17632/rvs6gf4sn2.4
EJKL_Linux64.zip	Binary executable file of the software for Linux 64-bit systems	CERN-OHL-S	http://dx.https://doi.org/10.17632/rvs6gf4sn2.4
EJKL_Win64.zip	Windows installer for the software	CERN-OHL-S	http://dx.https://doi.org/10.17632/rvs6gf4sn2.4
EJKL_software_source.zip	Source code of the software for LabVIEW 2017 or newer version	CERN-OHL-S	http://dx.https://doi.org/10.17632/rvs6gf4sn2.4
EJKL_software_manual.odt	EJKL PC software manual	CERN-OHL-S	http://dx.https://doi.org/10.17632/rvs6gf4sn2.4
example.ejkl	Example sequence used for testing the device	CERN-OHL-S	http://dx.https://doi.org/10.17632/rvs6gf4sn2.4

## Bill of materials

Table 1 lists all the necessary components of the device, their prices, names, and the signatures defining these elements precisely and used to identify them on PCBs. All electronic components on PCBs are assembled using through-hole technology (THT). Links to online shops where the elements can be ordered are also included in the table. A more extensive ODS file containing exact links to all components can be found in the download files.

**Table 1:** Bills of materials.

Component	Symbol	Number	Cost per unit, €	Total cost, €	Source of materials	Material type
Supercapacitors board	Not applicable	1	43.86	43.86	www.merkar.pl^*,**^	PCB
PWM board	Not applicable	1	20.0	20.0	www.merkar.pl^*,**^	PCB
Power transistors board	Not applicable	1	37.48	37.48	www.merkar.pl^*,**^	PCB
Drivers board	Not applicable	1	24.57	24.57	www.merkar.pl^*,**^	PCB
Power supply board	Not applicable	1	26.75	26.75	www.merkar.pl^*,**^	PCB
Connector board	Not applicable	1	22.60	22.60	www.merkar.pl^*,**^	PCB
Zener diode 2.7 V	BZX55C2V7	20	0.04	0.8	www.tme.eu	Electronics
Zener diode 12 V	BZX55C12	15	0.04	0.6	www.tme.eu	Electronics
Fast diode	DSEI120-12A	4	4.65	18.6	www.tme.eu	Electronics
Diode	P2500B	3	0.7	2.1	www.tme.eu	Electronics
Diode bridge	KBPC804	2	0.7	1.4	www.tme.eu	Electronics
Supercapacitor	DB5U207M30045HA	20	8.06	161.2	www.tme.eu	Electronics
Capacitor 2.2 nF	C322C222J1G5TA	14	0.18	2.55	www.tme.eu	Electronics
Capacitor 470 μF	KM 470U/63V	6	0.29	1.74	www.tme.eu	Electronics
Capacitor 10 μF	KM 10U/63V	6	0.08	0.5	www.tme.eu	Electronics
Fuse holder	KEYS3557-2	8	0.91	7.28	www.tme.eu	Electronics
Fuse 40 A	0287040.PXCN	1	0.09	0.09	www.tme.eu	Electronics
Fuse 35 A	0287035.PXCN	4	0.09	0.37	www.tme.eu	Electronics
Fuse 5 A	0287005.PXCN	3	0.09	0.28	www.tme.eu	Electronics
Resistor 10 kΩ, 0.25 W	CFR0W4J0103A50	44	0.01	0.53	www.tme.eu	Electronics
Resistor 1 kΩ, 0.25 W	CFR0W4J0102A50	36	0.01	0.43	www.tme.eu	Electronics
Resistor 100 Ω, 0.25W	CFR0W4J0101A50	12	0.01	0.14	www.tme.eu	Electronics
Resistor 0.56 Ω,10 W	PRW0AWJW56KB00	2	0.22	0.44	www.tme.eu	Electronics
Resistor 10 Ω, 300 W	HS300 10R F	1	27.41	27.41	www.tme.eu	Electronics
N-MOSFET	BS170	16	0.08	1.28	www.tme.eu	Electronics
N-MOSFET	IRF530N	1	0.7	0.7	www.tme.eu	Electronics
P-MOSFET	IRF5210	17	1.28	21.76	www.tme.eu	Electronics
NPN transistor	2N6292	2	0.69	1.38	www.tme.eu	Electronics
Thyristor	BT152	1	0.7	0.7	www.tme.eu	Electronics
Voltage regulator	L7805	1	0.7	0.7	www.tme.eu	Electronics
Timer	NE555	5	0.25	1.25	www.tme.eu	Electronics
Digital resistor	DS1804-010+	4	2.59	10.36	www.tme.eu	Electronics
Logic gate	74LS08	1	0.67	0.67	www.tme.eu	Electronics
Microcontroller	Arduino UNO	1	17.80	17.80	www.tme.eu	Electronics
MOSFET driver	TC4427A	4	1.01	4.04	www.tme.eu	Electronics
LED 3 mm blue	L-7104PBC-A	1	0.43	0.43	www.tme.eu	Electronics
LED 3 mm red	L-7104SRC	5	0.14	0.7	www.tme.eu	Electronics
LED 3 mm green	L-7104CGCK	1	0.14	0.14	www.tme.eu	Electronics
LED 3 mm yellow	LL-304UYC4B-Y2-2BD	1	0.14	0.14	www.tme.eu	Electronics
Transformer	TMB100/36V	1	23.7	23.7	www.tme.eu	Electronics
DIP 8 socket	2-1571552-2	13	1.56	20.28	www.tme.eu	Connectors
DIP 14 socket	2-1571552-3	1	2.02	2.02	www.tme.eu	Connectors
PCB terminal block ways: 3	TB-5.0-K45-3P	1	0.82	0.82	www.tme.eu	Connectors
PCB terminal block ways: 2	TB-5.0-K45-2P	27	0.33	8.91	www.tme.eu	Connectors
Pin header 1 × 20	68000-220HLF	2	0.75	1.5	www.tme.eu	Connectors
IDC connector 1 × 10	DS1013-10SSIB1	2	0.1	0.2	www.tme.eu	Connectors
IDC connector 1 × 20	DS1013-20SSIB1	2	0.33	0.66	www.tme.eu	Connectors
IDC cable	FC10150-S	1	1.03	1.03	www.tme.eu	Connectors
IDC cable	FC20300-S	1	2.01	2.01	www.tme.eu	Connectors
Power socket	PX0580/63	1	1.22	1.22	www.tme.eu	Connectors
BNC socket	1-1337452-0	2	4.80	9.60	www.tme.eu	Connectors
Female audio connector	NL4MP	8 (4 on panel+1 per coil)	2.12	16.96	www.tme.eu	Connectors
Male audio connector	NL4FC	8(2 per coil)	6.66	26.44	www.tme.eu	Connectors
Tip: ring M6	R 8-6 NICHIFU	10	0.16	1.6	www.tme.eu	Connectors
Tip: ring M6	19323-0014 MOLEX	10	0.4	4.0	www.tme.eu	Connectors
Tip: ring M3	19324-0002 MOLEX	4	0.22	0.88	www.tme.eu	Connectors
Terminal: flat; 6.3 × 0.8 mm	VDADF5.5-250A JST	6	0.14	0.84	www.tme.eu	Connectors
Coil wire	DN1E1.00/0.50 BQ	1	17.75	17.75	www.tme.eu	Copper wire
Soldering wire	BROFIL 63 B2.1 0.5MM 100G	1	5.45	5.45	www.tme.eu	Tin/lead
Loudspeaker cable; 4 × 2.5 mm^2^	400092 HELUKABEL	6 m	3.94	23.64	www.tme.eu	Cable
Cable 10 mm^2^, red	4520045 LAPP	5 m	2.40	12.0	www.tme.eu	Cable
Cable 10 mm^2^, black	4520015 LAPP	5 m	2.40	12.0	www.tme.eu	Cable
Cable 2.5 mm^2^, red	64143 HELUKABEL	5 m	0.91	4.55	www.tme.eu	Cable
Cable 2.5 mm^2^, black	64139 HELUKABEL	5 m	0.86	4.30	www.tme.eu	Cable
Cable 2.5 mm^2^, green/yellow	64140 HELUKABEL	5 m	0.89	4.45	www.tme.eu	Cable
Cable 2 × 1 mm^2^, red/black	C102-1.00 TASKER	5 m	0.86	4.30	www.tme.eu	Cable
Plug cable	SN311-3/07/1.8B	1	3.26	3.26	www.tme.eu	Cable
Set of heat shrink sleeves	CB-HFT-SET1 CYG	1	5.95	5.95	www.tme.eu	Polyolefin
Rocker switch dpst 30 × 22	R210-1-C5G-BR9NWC	1	1.39	1.39	www.tme.eu	Button/switch
Momentary push button	PBS33	1	0.65	0.65	www.ebay.com	Button/switch
Analog ammeter	HD-50-5A	1	2.81	2.81	https://botland.store/voltage-indicators/10201-analog-ammeter-panel-dh-50-5a-5904422315153.html	Meter
Analog voltmeter	85C1 0-50V	1	4.97	4.97	www.ebay.com	Meter
Case	M6019305	1	153.44	153.44	https://de.farnell.com	Metal
Front panel	M6019001	1	21.98	21.98	https://de.farnell.com	Metal
Case mounting kit	M6000035	1	5.74	5.74	https://de.farnell.com	Metal
Glass/epoxy sheet		1	17.25	17.25	https://kontakt-sa.pl/plyty-szklano-epoksydowe/4865-plyta-szklano-epoksydowa-50x1000x1000mm-kg.html^**^	Glass/epoxy
Filament Noctuo ABS 1.75 mm, 0.75 kg		1	17.40	17.40	https://botland.com.pl	ABS
Screw M3 cheesehead 10 mm	DIN 7985 A2 M3x10	16	0.01	0.16	https://bejmet-nierdzewne.pl	Steel
M3 countersunk 10 mm	DIN 965 A2 M3x10	14	0.01	0.14	https://bejmet-nierdzewne.pl	Steel
M3 countersunk 20 mm	DIN 965 A2 M3x20	27	0.01	0.27	https://bejmet-nierdzewne.pl	Steel
M3 countersunk 8 mm	DIN 965 A2 M3x8	8	0.01	0.08	https://bejmet-nierdzewne.pl	Steel
M4 countersunk 16 mm	DIN 965 A2 M4x16	8	0.01	0.08	https://bejmet-nierdzewne.pl	Steel
M5 cheesehead 60 mm	DIN 84 A2 M5x60	8	0.07	0.56	https://bejmet-nierdzewne.pl	Steel
M5 cheesehead 10 mm	DIN 84 A2 M5x10	4	0.02	0.08	https://bejmet-nierdzewne.pl	Steel
M6 hex head 80 mm	DIN 933 A2 M6x80	4	0.1	0.4	https://bejmet-nierdzewne.pl	Steel
M3 nut	DIN 934 A2 M 3	65	0.005	0.33	https://bejmet-nierdzewne.pl	Steel
M4 nut	DIN 934 A2 M 4	8	0.007	0.06	https://bejmet-nierdzewne.pl	Steel
M5 nut	DIN 934 A2 M 5	12	0.01	0.12	https://bejmet-nierdzewne.pl	Steel
M6 connector nut	DIN 6334 A2 M6	12	0.16	1.92	https://bejmet-nierdzewne.pl	Steel
			Total:	942.19		

*The service company which manufactured the item. One can find a company nearby that provides similar services.

^**^The website available in Polish. One can find a store nearby that provides the same items.

## Build instructions

### Safety issues

The device described here is a high-power one involving potential risks associated with its construction and usage. Although the applied maximum voltage of 50 V, under dry conditions, is generally not considered life-threatening, it is unpleasant and may be painful. Attention should also be paid to the high currents generated in the system, which may cause the elements within it to heat up to harmful temperatures, leading to burns and/or fire. Hence, **caution should be taken when constructing this device, and the entire assembly and operating manual should be read before its assembling and use.** The following general points should be remembered:.

i) The energy stored in supercapacitors can reach up to 10 kJ and can be released soon after a short circuit. It is enough to melt the paths on PCBs and heat 100 g of copper (coil weight) to about 200C. Therefore, the electronic elements can be assembled and repaired only when the supercapacitors are discharged. Any loose parts inside the installed device can lead to a short circuit (as screws) and must be removed.
ii) The force exerted on the moving core of the coil during pulses can cause high acceleration of elements (over 1000 m/s^2^). As the fast-moving core can hit and cause injury, it is necessary to ensure that during the pulse there is no one in the line of the coil axis, and hands should be placed away from the moving parts.
iii) In the event of an accidental short circuit, the device should always be electrically grounded during operation.

Other safety considerations relating to specific parts of the device are described in the relevant subsections of assembly and operating instructions. Finally, for safe use of the device, it is important to carry out a series of tests in the correct sequence and as per the provided protocol. If an element does not work as expected, it should be replaced in the device.

### PCB manufacturing

The best option for PCB manufacturing is to outsource the production to an external company providing services in this field. The gerber files included in the prepared package should be sent to the company. The recommended parameters for PCB manufacturing are:.

PCB material: FR4.
PCB thickness:1.5 mm.
Copper thickness: 105 μm (power transistors board, supercapacitors board), 35 μm (other boards).

### Soldering components on PCBs

**Fig. 3:**
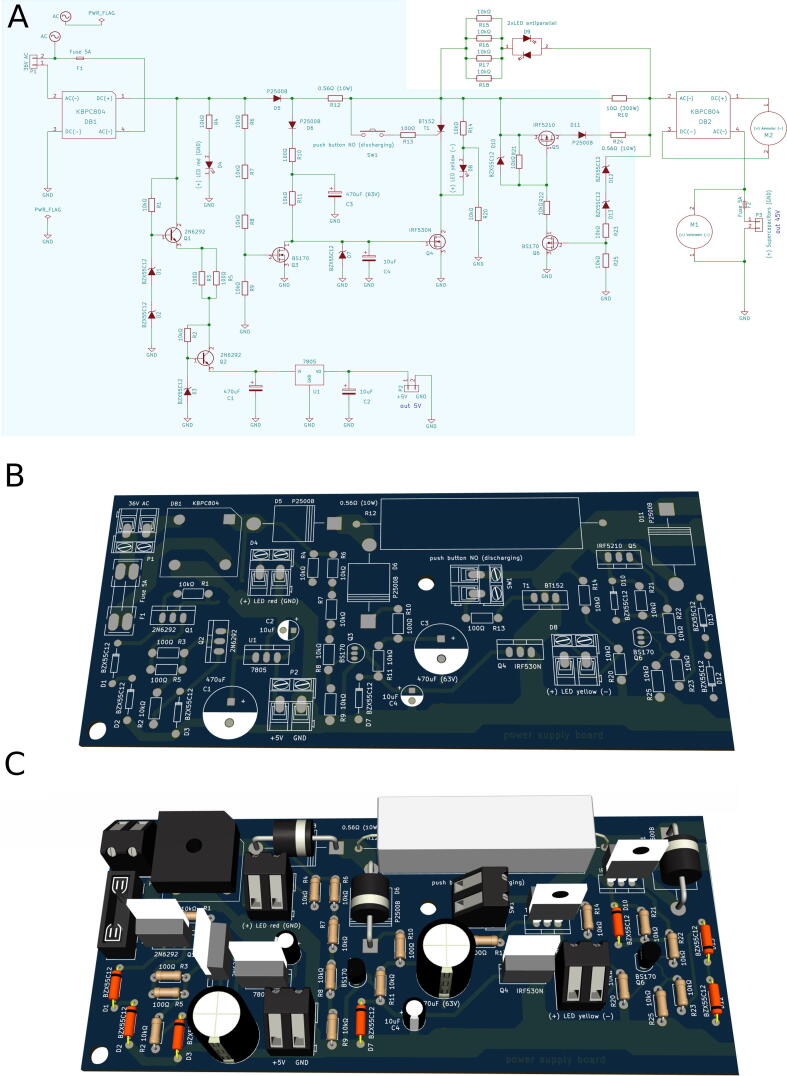
An example of a part of the power supply board with the footprints and description of the components on the board. A) Scheme of the board. A part presented in panels B and C is marked as blue frame. B) Board before soldering with visible footprints and descriptions. B) The same board with the components. The footprints and descriptions of transistors (designated on board as Q), diodes (designated on board as D), and capacitors (designated on board as C) are presented. (For interpretation of the references to color in this figure legend, the reader is referred to the web version of this article.)

Electronic components of PCBs can be easily made by hand. Footprints and signatures of all the elements on the boards are available, which clearly indicate how the elements should be soldered to the PCB. However, in case of doubt, one can check the scheme of circuit or (if one is not familiar with electronic symbols) the graphics related to 3D visualizations of PCBs included in the EJKL_3D_boards.zip package or use interactive preview of a board in KiCad software. Fig. 3 presents a part of power supply board as an example of how to interpret the schemes and footprints.

Elements located outside a given board as well as connections to other boards are marked with footprints of terminal block along with descriptions. The example presented in Fig. 3 applies to elements P1 (36 V input from transformer), P2 (5 V output for PWM board), SW1 (push button for discharging), and D4 and D8 (indicator light-emitting diodes (LEDs)).

The PWM board and drivers board have integrated circuits (designated by letter U). During PCB visualization, these components are soldered directly to the boards. However, it is better to use dedicated DIP sockets for easy replacement of components.

It is necessary to check the polarity of outputs from the terminal which are signatured (+) and (−) or (GND). In the case of LEDs, output (GND) or (−) should be connected with cathode marked by a shorter wire and (+) with anode marked by a longer wire. Other polarized elements have the descriptions of outputs and inputs. If information about the polarity of the terminal/element is not provided, it means that it does not matter how the element is connected.

In this project, we used a number of MOSFETs that are sensitive to electrostatic discharge, and hence, soldering is not recommended if one uses a synthetic sweater as there is a risk of accidental spark, which can damage the semiconductor components. It is also recommended to use a resistance instead of a transformer soldering iron.

### Mounting board

The mounting board can be cut from a glass/epoxy sheet. It can be manufactured by hand using a drill and jigsaw, but a better option is to obtain it from CNC service. Stl and OpenSCAD files are available for download.

M3 (20 mm) countersunk screws, M3 nuts, and spacer sleeves are required to mount PCBs to the mounting board, and M4 (16 mm) countersunk screws and M4 nuts for mounting the transformer and 300-W resistor. Spacers can be made on a 3D printer (23 copies) using virtually any plastic material. OpenSCAD and stl files are available for download. The sleeves can also be made by hand, but they should have the appropriate length to ensure that the pieces fit into the holes in the case at the end. Fig. 1 illustrates the mounting of the elements to the board.

### Wires

**Fig. 4:**
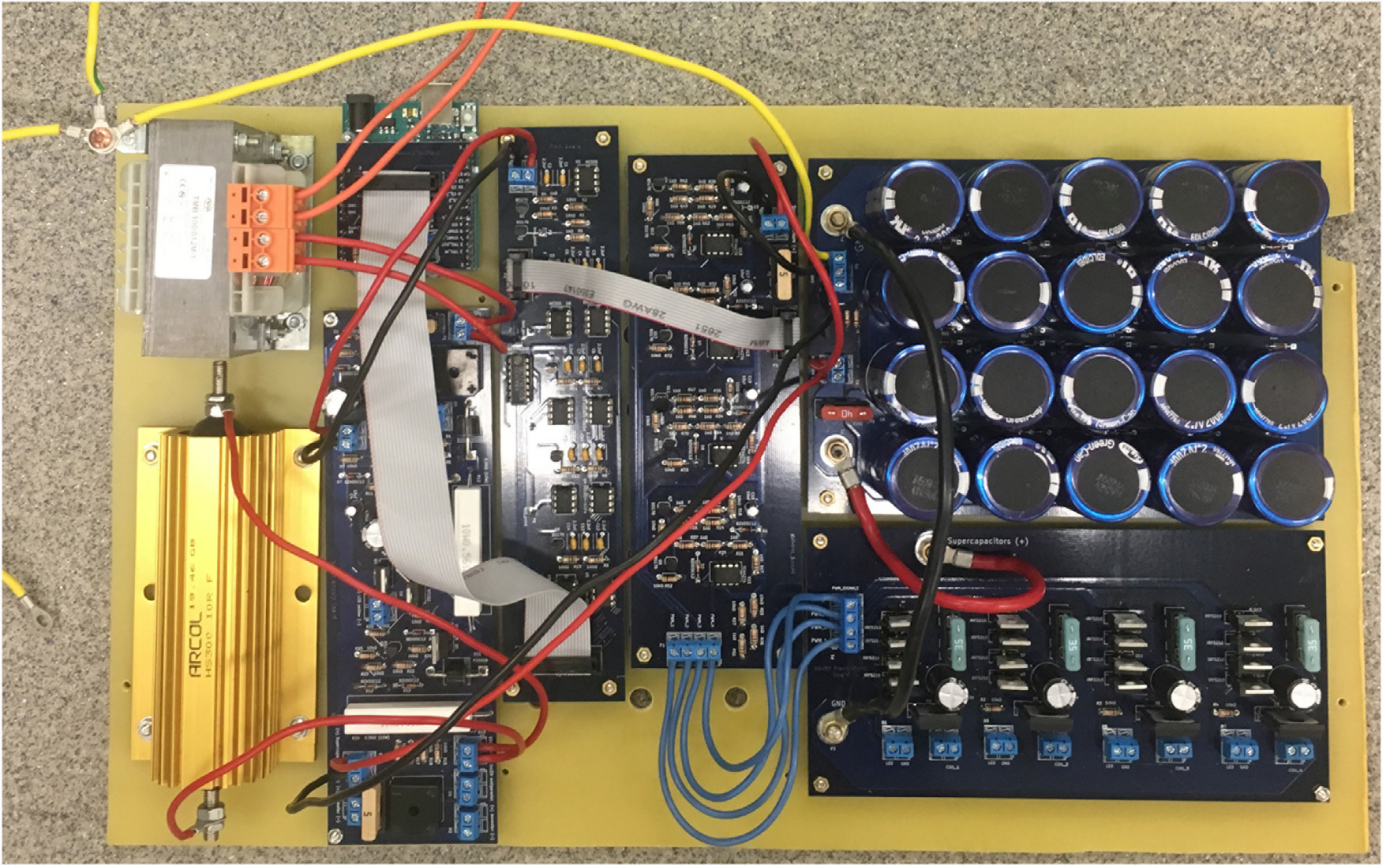
A snapshot showing the electronic parts mounted on the boarding plate.

Elements on the mounting board should be connected to each other through wires according to the description of the terminal blocks on the boards. A schematic representation of wiring is shown in Fig. 2, Fig. 4.

The supercapacitors board and transistors board should be connected with a 10-mm^2^ wire tipped with an M6 ring (R 8–6 NICHIFU). The rings should be screwed to the pads on the boards using an M5 10-mm screw. The Arduino, PWM board, and drivers board should be connected with ribbon cables, which is possible only with the use of a 2.5-mm^2^ cable for other connections between the boards. It is a good practice to tin the tip of wires put in terminal blocks. For 300-W resistor, an M6 ring (19323–0014 MOLEX) should be used as a tip.

ATTENTION: The power supply board must be connected to the low-voltage output of the transformer (usually connected to a thicker winding). Incorrect transformer connection results in an output voltage of over 1000 V which can destroy the device and may also be life-threatening. The transformer input wires should be terminated with a flat terminal 6.3Χ0.8 mm in order to connect them to the power socket (PX0580/63).

The ground (GND) should be connected to the transformer, supercapacitors board, and case (yellow-green cables shown in Fig. 4). One wire should be connected to power socket and terminated with a flat tip. The second wire is ended with a metal ring and connected to the case.

The LEDs, ammeter, voltmeter, and BNC (Bayonet Neill-Concelman) socket should be connected to the terminal blocks using a 1-mm^2^ cable. To connect the meters, the cables must be terminated with M3 rings. The pins of the LEDs should be soldered to wires and isolated using heat shrink sleeves. In the case of the terminal described “2Χ LED antiparallel” on the power supply board, two LEDs should be connected parallel but in opposite directions. These LEDs indicate charging and discharging of the supercapacitors. In this project, we used blue and green diodes, while red LEDs were used to indicate the voltage on the coils.

**Fig. 5:**
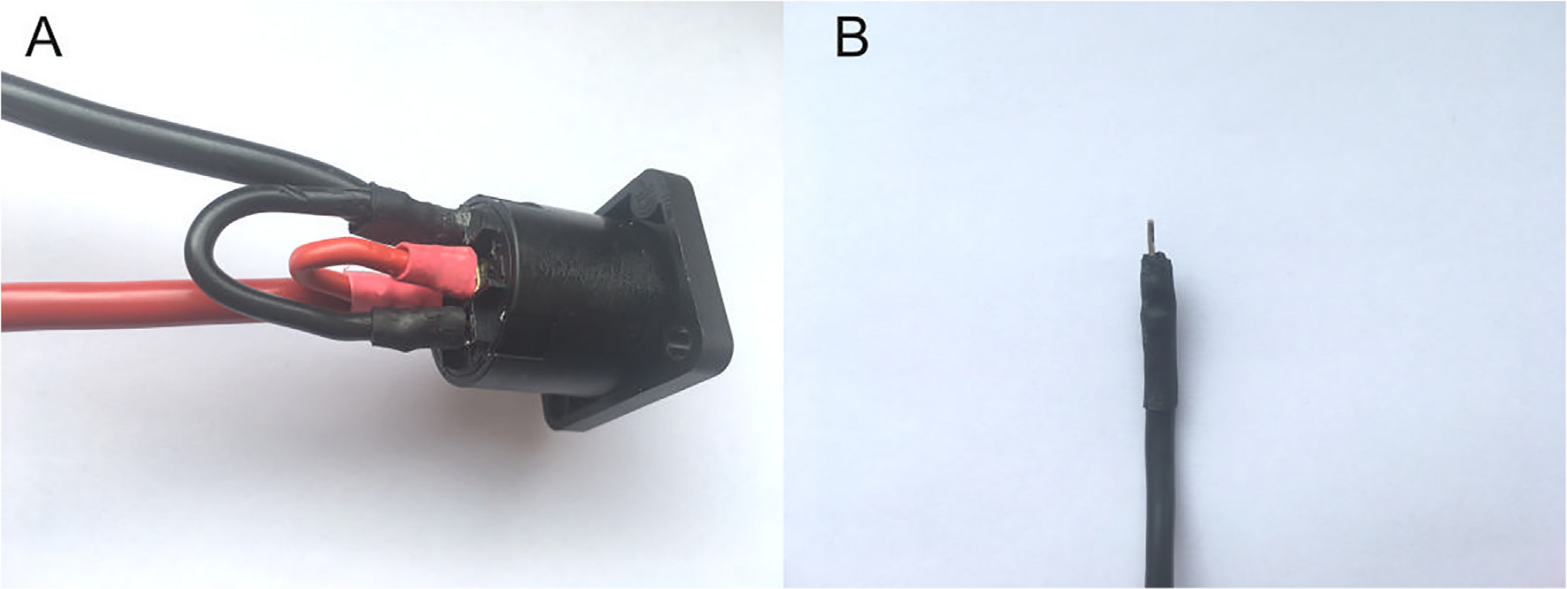
A) Picture of the bridged pins of the female audio socket. B) Tip of 10-mm^2^ cable adapted to the terminal block.

The pins of female sockets of all audio connectors (NL4MP) should be bridged using a 2.5-mm^2^ cable. Pin 1 + should be connected to pin 2+, and pin 1− to pin 2− (labels are provided on the sockets). For sockets mounted on the panel, current to the pins should be passed through a 10-mm^2^ wire. Pins should be isolated via heat shrink sleeves. Fig. 5A shows the socket prepared as described. In the case of sockets mounted in the coil holder, the pins should not be isolated, because the coil wires must be soldered to them. The 10-mm^2^ cable that connects the terminal block to the audio socket is too thick to fit the terminal block. Therefore, a short winding wire with removed enamel should be soldered to the end of the cable as a tip and also insulated with heat shrink sleeves (Fig. 5B).

### Coils, coil holder, and connecting cable

**Fig. 6:**
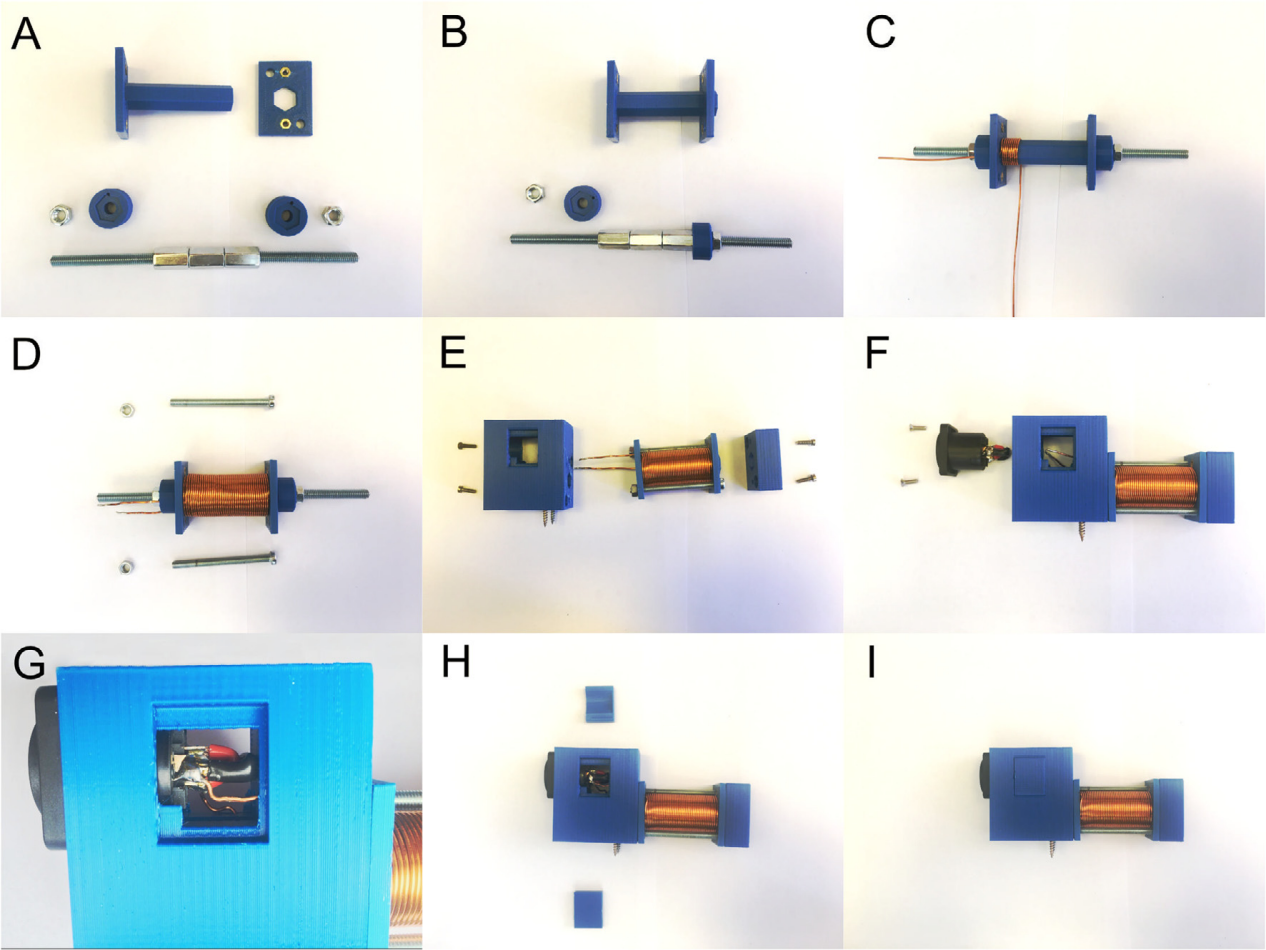
A sequence of photos showing step-by-step process of assembly of the coils.

To wind and mount the coils, the spool elements and the coil holder should be printed using a 3D printer. The printout should be made of ABS with completely filled interior of the elements. The ABS material was chosen for several reasons: it is widely used in the 3D printing technology, durable, and has a relatively high melting point. In addition, the ABS components can be easily glued or modified to some extent with acetone if damaged. However, any diamagnetic and durable material can also be used instead of ABS. OpenSCAD and stl files for all the elements are available for download. A step-by-step assembly process of the whole coil and the coil holder is shown in Fig. 6.

Before assembling and winding the coil, the M3 nuts must be inserted into the dedicated holes in the spool rims. The holes should be slightly tight, so the nuts must be pressed into the right place using a force. A vice can be used for this purpose. The spool rim on the spool with the nuts should be placed on the inside and temporarily stabilized to prevent accidental disassembly during winding. It can be done by putting the M6 threaded rod with twisted M6 connector nuts through the center of the coil and block using dedicated blockers (Fig. 6A and B). OpenSCAD and stl files for 3D print of blocker are available for download. A winding copper wire of a 1-mm diameter should be used to wind the coil. The wire should be threaded through the holes in the rim closer to the center of the spool, leaving about 10 cm of wire protruding outside the rim. Exactly six layers of winding should be wrapped, and the wire should be threaded through the second hole in the rim. The number of layers and the diameter of the wire on the coil were calculated to obtain the highest possible magnetic field. The maximum impulse current, which is safe for the batteries of the supercapacitors and the power transistors, is about 100 A. As the coil operates at a voltage of about 45 V, its static resistance should be about 0.4 Ω. The coil’s static resistance depends on the length and diameter of the winding wire. The larger the diameter of the wire, the longer the length of the wire and the more the turns in the coil for the same current. However, the coil dimensions will also increase. Due to the PWM control, which causes voltage oscillations, the skin effect becomes significant, indicating that increasing the diameter of the conductor does not cause a reduction in resistance in the square, but in extreme cases, in the first power. At 20 kHz, the maximum diameter of the copper wire at which the skin effect can be ignored is ∼1 mm. This implies that if a conductor with a diameter of greater than 1 mm is used, the gain in the number of turns will be smaller, and thus the magnetic field strength, in relation to the increase in the size of the coil. Considering the above information and the copper resistivity, the length of the copper wire (1 mm) in the coil should be about 17–20 m. For a 5-cm-long coil with an internal diameter of about 1.5 cm, the optimal wire length is achieved with six layers of winding. The wire should be wound in the direction corresponding to the increase in the thickness of the ramp, starting from the hole. Although the wire can be wound by hand (Fig. 6C), it is better to put the spool in a lathe and wind the wire at low revs. After winding, the coil must be screwed on using two M5 60-mm screws (heads of the screws must be placed on the opposite side of the coil to the protruding wires) (Fig. 6D). At this stage, the temporary stabilization of the coil can be disassembled. Before the coil is placed in the holder, enamel should be removed from the protruding wires using a file. The screws for mounting the coil to the base should also be placed in the vertical mounting holes of the holder where the socket will be located. The coil should be screwed to the holder with M3 10-mm screws, and the wires should be led to the socket cavity (Fig. 6E and F). The socket with properly bridged pins (described in the “Wires” section) should be inserted, soldered to wires, and screwed into the holder.

The wire extending from the outer part of the winding should be soldered to the external pins of the socket (1− and 2−), and the wire extending from the inner part of the winding should be soldered to the internal pins (1+ and 2+). It is important to ensure appropriate soldering of the wires to the pins to avoid accidental contact and short circuits (Fig. 6G). Finally, the socket cavity should be plugged with dedicated elements (Fig. 6H and I).

The connection cable should be a four-core speaker cable with two male audio connectors (NL4FC) with a length of 1.5 m. Male connectors should be installed as per the manufacturer’s instructions. Pins with the same label should be connected on the opposite side of the cable. Be careful not to allow a crossover cable to form in the audio connection as this will cause a short circuit at high current during the pulse.

### Piston

The piston consists of two parts: metal core and a plunger printed on a 3D printer. The printout of the plunger should be made with ABS with complete filling of the interior of the elements. OpenSCAD and stl files for all elements are available for download. The core can be made by screwing three connector nuts onto an M6 80-mm screw. The walls of the nuts should form one plane. The rest of the thread should be screwed into the printed plunger. The precisely screwed piston should move without much resistance in the spool hole.

### Tests and power tuning

After soldering and before completely wiring the device and connecting it to the power supply, certain control measurements should be taken with a multimeter. This is important to minimize the risk of uncontrolled high-current short circuits during the test connection of the equipment to the power supply resulting from incorrect soldering. The following three steps should be performed before connecting the PCBs.

• Short circuit between the gates of power transistors and their supply must be checked. The resistance between the PWM signal inputs (P1 terminals) and + and GND pads (P2 and P3) on the power transistors board should be in theory infinite (over MΩ). A much lower resistance indicates that one of the power transistors in the channel is damaged and requires replacement.
• The fast diodes on the power transistors board must be checked. When the positive terminal of the multimeter (red) is connected to the positive terminal in the coil supply terminal block and the negative terminal of the multimeter (black) is connected to the GND of the coil terminal block, the diode should be reverse-biased. The multimeter should show open circuit. When the multimeter is connected to coil terminal block in opposite directions, the diode should be forward-biased. The voltage drop should be between 200 and 1800 mV.
• The resistance between any pins of the R12 resistor and the ground on the power supply board should be checked and ensured to be around 20 kΩ. A much lower resistance indicates that thyristor (T1) is damaged.

The next test should be done when the boards are already mounted on the mounting board but are not fully wired. To perform this test, the device should be connected to the power. It is important to turn off the power when the device is prepared for the next test.

• The transformer and power supply board must be connected. The connection of the transformer should be checked (as described in the “Wires” section), and the rest of the boards should not be connected for this test. Power must be turned on, and the output voltage in the P2 terminal block should be about 5 V.
• The PWM board should be connected to the power supply board and Arduino (Fig. 2, Fig. 4). The grounding must be temporarily connected to terminal (GND) in the “supercapacitors” terminal block (P3) on the power supply board. The PWM board and its calibration should be checked with the PC software and an oscilloscope. Pin 3 provides a preview of a clock that should generate pulses of about 4 μs, separated by about 50 μs. Pins 4–7 allow previewing the PWM signal that will be applied to the coils during pulses. The width of the pulses corresponds to the output power provided to the coil. Coil power calibration in the software is done by determining the minimum value of the parameter “max power value” (C2 in the software manual), at which the signal is fully filled with the pulses. For power calibration, the firmware should be uploaded to the Arduino microcontroller and the provided software is installed on a PC according to the software manual. Firmware installation is easy and is described in Arduino tutorials available online. The firmware is also included in the repository files attached to this paper. However, calibration is not absolutely necessary, because the initial error of the parameter “max power value” should not be greater than 15%. As the minimum nonzero duty cycle is about 10%, some discrepancies in the settings of “power” and the duty cycle may possibly appear at low “power” values.
• The drivers board must be connected to the assembly from the previous test. Two 9-V batteries should be prepared, connected in series, and fixed to the drivers board instead of the supercapacitors. The batteries should not be connected with the power supply board. Voltage must be measured at the four outputs of the drivers board (P1). If no pulse is applied to the channel, then the voltage between the corresponding output in the P1 terminal block and the drivers board supply (+in P2) should be about 0 V. When pulse with full filling (100% power) is given to the channel, the meter connected in the same way should show a difference of about 10–12 V. All channels should be checked. If any channel behaves differently than it should, then it is necessary to check whether the zener diodes, transistors, and driver are mounted correctly.

If the system has passed all the above tests, wiring of the device can be completed as shown in Fig. 2, Fig. 4. After connecting all the wires and elements, the whole electronics should be tested before they are placed in the case. The charging/discharging process, as well as the operation of power transistors, should also be checked. Although not necessary, it is recommended to perform an additional preliminary charging/discharging test before the power transistors board is connected. Preliminary testing of power transistors board does not require the coils to be connected to the output. However, a test can also be carried out with the coil-loaded output, so the cable sockets can be better connected to appropriate terminals before testing the power transistors board.

• Charging should start immediately after the device is switched on. The charging current should range between 2 and 3 A but it decreases slowly until the voltage on the supercapacitors reaches 25 V. After 25 V is reached, the charging current increases to 4 A and then gradually decreases to 0 A. The final voltage on the supercapacitors should be around 48 V. The charging process is indicated by one of the LEDs in the pair of antiparallel diodes (originally by the blue one). At the time of charging, the transformer, 300-W resistor (R19), 0.56-Ω resistors (R12, R24), diode bridges (DB1, DB2), transistor IRF5210 (Q5), and diodes P2500B (D5, D11) may become hot; however, the temperature should not exceed ∼ 80 °C. The other elements should not be allowed to warm up. In particular, thyristor T1, transistor Q4 on the power supply board, and power transistors (Q1–Q16) and diodes (D2, D4, D6, D8) on the power transistors board should remain at room temperature. Temperature checking of the components should not be done with hand, and a thermocouple should be used instead.
• When the power is turned off and voltage on the supercapacitors is not 0 V, the yellow LED should be on. Then, the discharging button should be pressed to begin the discharging process, which is indicated by the second diode (originally the green one) in the antiparallel pair. The discharging current should range between 4 and 5 A, and decreases gradually to 0 with the decrease of the voltage on the supercapacitors. At the time of discharging, the diode bridge (DB2), 300-W resistor, thyristor (T1), and transistor (Q4) may become hot, but the temperature of the other components (DB1 diode bridge, diode D5, and resistor R12 and transistor Q4 on the power supply board) should not be higher than that during charging.
• At the beginning of testing the power transistors board, the LEDs should be off and the voltage on the coil terminal block should be 0 V when no pulses are applied to a given output. If the LED is on under these conditions, the device should be turned off immediately and the supercapacitors must be discharged by pressing the discharge button. Then, the investigated channel from the PWM terminal block must be disconnected and the resistance between the signal terminal and ground should be checked according to the above-described procedure. By contrast, when a pulse is applied, the voltage on an investigated terminal block should be close to that on the supercapacitors. If the pulse is fully filled, the meter should show ∼ 45 V, and the LED should light up.
• To test with a loaded output, the coil should be connected with the appropriate cable to the socket (described in the “Coils, coil holder, and connecting cable” section). Pulses of 500 ms should be applied with different degrees of filling (10%, 50%, 100%) at intervals of 5 min to allow a coil to cool down. The temperature of the transistors and diodes should not be more than 150 °C, and it is necessary to check that the elements are not desoldered from PCB during the pulse.

For a better understanding, a video showing the wiring of boards and electronics tests is provided in the pictures_movie.zip package.

### Case

After all the electronic parts are mounted on the mounting board, wiring is done and electronics tests are performed, following which the electronics can be placed in the case. However, before placing, the mounting holes should be prepared in the front and back panel of the case. OpenSCAD and stl files for both front and back panels are available for download. As it is strongly recommended to make holes in the panels using a milling machine, the best option is to outsource the manufacturing of panels to a company that provides such services.

First, all the sockets, buttons, and meters should be mounted on the panels. The LEDs and all cables that are connected to the elements on the panels and not soldered to these elements should be screwed into the terminal blocks. Then, the entire mounting board should be inserted in such a way that the ground can be screwed to the metal parts of the case and the ground wiring should enter through the cutout in the mounting plate (upper right corner in Fig. 1). Next, the mounting board should be screwed to the chassis using M3 countersunk 10-mm screws and grounding wire should be connected to the case. The diodes should be tightly inserted into the holes in the panels, and the cables should be connected to the buttons and meters. The BNC sockets with the soldered cables should be placed on the back panel and connected to the terminal blocks on the Arduino connector board. The external pins (1– and 2–) of the audio socket (Fig. 5, black wire) should be connected with grounding (GND). The cables soldered to the audio sockets should be placed in the terminal blocks in such a way that pins 1– and 2– are connected to the terminal labeled GND. When all the components and cables are connected, the front and back panel can be screwed to the case using a specific mounting kit.

## Operation instructions

### Operating hardware

The device operation requires a PC with dedicated software which is available for download. The installation and operation instructions for the software can be found in the attached manual. The steps for the device operation are described below.

1) The device should be turned on, and the supercapacitors must be allowed to be charged fully. Charging becomes complete when the voltage on the supercapacitors reaches about 45 V and the indicator LED is off which takes a few minutes. Charging can also be checked by a voltage indicator in the software.
2) The software should be run, the device should be connected to the available USB port, and connection should be established between PC and the Arduino controller (this step is explained in the attached software manual). Then, the power of the coils and the sequences must be set according to the software manual.

A considerable amount of heat is released during pulses at the power amplifier and the coils. Therefore, it is critical to use pulses that are not longer than needed to avoid overheating of the elements. Otherwise, the released heat may damage the device and/or melt the plastic spool of the coils. However, our tests revealed that even complete discharging of the capacitors through the coil did not affect the electronics, despite a significant overheating of the power amplifier, but the heated wire of the coil caused deformation of the spool. Taking into account the technical parameters of the coil and the softening point of ABS material, the safe pulse time t_safe_ can be estimated as follows for a given duty cycle expressed as a percentage of maximum power (“power” sliders software):.

t_safe_ < 18.3 [JΩ/K]*(T_softening_ − T_initial_)/(U^2^*duty_cycle/100) [K/V^2^],

where U is the voltage on the supercapacitors. The 18.3 JΩ/K coefficient is the product of the density, specific heat and resistivity of copper and the square of the length of the wire wound on the coil. T_softening_ and T_initial_ are the temperature at which the plastic material becomes soft and the initial temperature of the coils, respectively [either in Kelvin or Celsius]. Assuming that T_softening_ and T_initial_ are 80C (for ABS) and 20C, respectively, and U is 45 V, the equation can be simplified to:.

t_safe_ < 50/*DutyCycle* [s].

It means that 0.5 s is a practical limit of the full-power pulse.

3) The initial position of the plunger should be set inside the core of the coil. It should be noted that the maximum force of piston is obtained when half of the metal core of the plunger is pushed inside the coil. When a pulse is applied, the metal cores are pulled into the coil. When the center of the metal core of the piston and the center of the coil are aligned, no force acts on the piston.
4) A designed sequence should be sent to the device, all mechanical parts should be ensured to be in the correct position, and the sequence should be run. It is important to maintain a safe distance from the plunger when the sequence is executed.
5) After finishing the sequence, the coil temperature must be checked. If it is too warm, the coils should be allowed to cooled down before the next sequence is executed.
6) After completing the work, the devices should be turned off and the discharge button should be pressed.

### Fuse replacement

If the coils stop responding (the indicator LED does not light up and the piston does not move), the coil fuse may be considered to be defective and should be replaced. In such cases, the device should be turned off and supercapacitors should be discharged. Then, the front panel should be screwed and the fuse should be checked. If the fuse is blown, it must be replaced with a new one and the device should be turned on.

If a coil does not respond after executing a sequence, the voltmeter shows 0 V after switching off the device, and the yellow LED does not light up after pressing the discharge button, the discharging process cannot be started and the fuse on the supercapacitors board may be blown and cannot be discharged. In such cases, the back panel should be unscrewed and the jumper should be shorted on the supercapacitor board, which is used for emergency discharge. For safety reasons, the device must be left as such for a couple of days (usually 2) to allow full discharging of the supercapacitors. After the discharging process, the fuse can be replaced and the device can be tested. The fuse should never be replaced without full discharging of the supercapacitors as it may cause burns.

### Validation and characterization

**Fig. 7:**
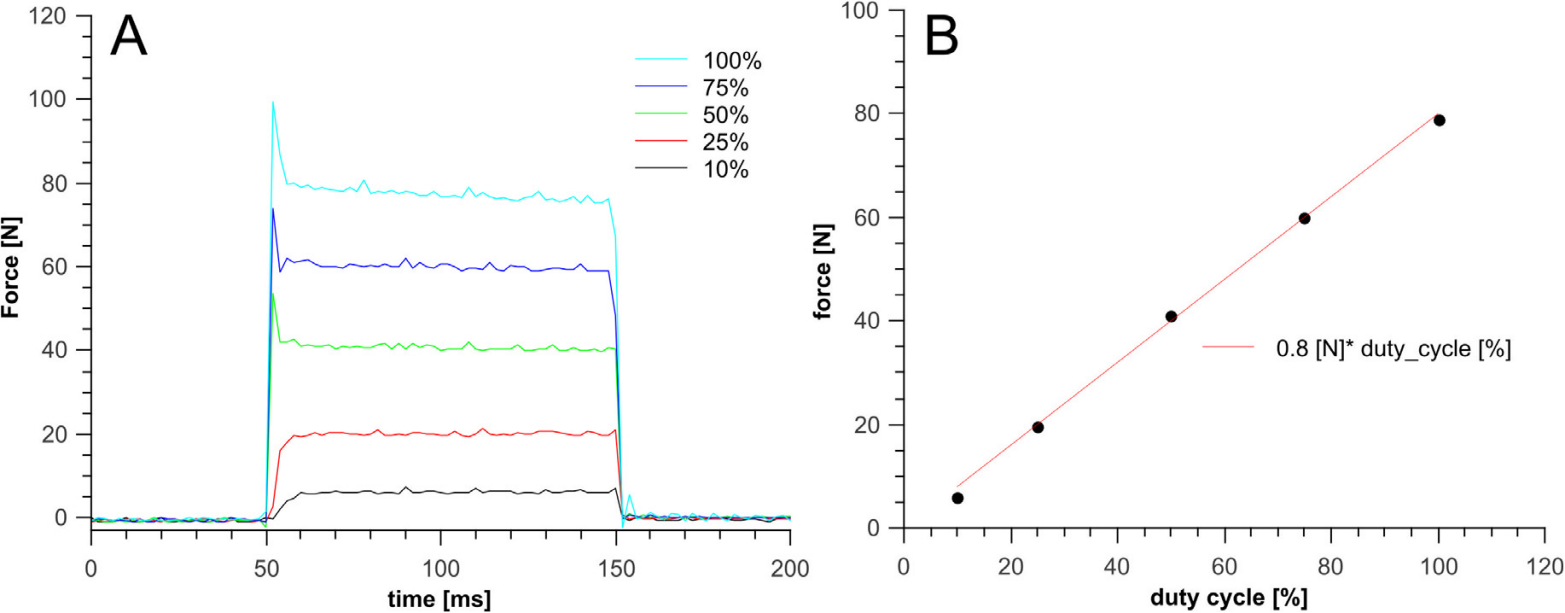
A) Force exerted on the piston during pulses of different duty cycles. B) Dependence of the force exerted by the piston on the duty cycle of the pulse.

To validate the device, a static force exerted by the piston was measured as a function of the percentage of the duty cycle (the “power” parameter adjusted through the software). The force was measured with a strain gauge beam. The description of the strain gauge is provided elsewhere [10]. The coil and the gauge beam were placed such that the metal part of the plunger core is halfway outside the coil (maximum force position) and 100-ms pulses with 100%, 75%, 50%, 25%, and 10% power settings were applied to the coil. Disturbance at the beginning and end of strong pulses shown in the force–time diagrams resulted from the decaying vibrations of the gauge beam after a rapid change in the force exerted on the beam. Fig. 7 presents the dependence of the piston force on the percentage of duty cycle of the pulses. As shown in the figure, the piston responds to commands in milliseconds, and a close linear correlation is found between the duty cycle and the force exerted by the piston. In addition, the current of the coil during pulse with 100% duty cycle was measured by a clamp meter. The measured values ranged between 100 and 120 A, which is in line with the theoretical predictions.

**Fig. 8:**
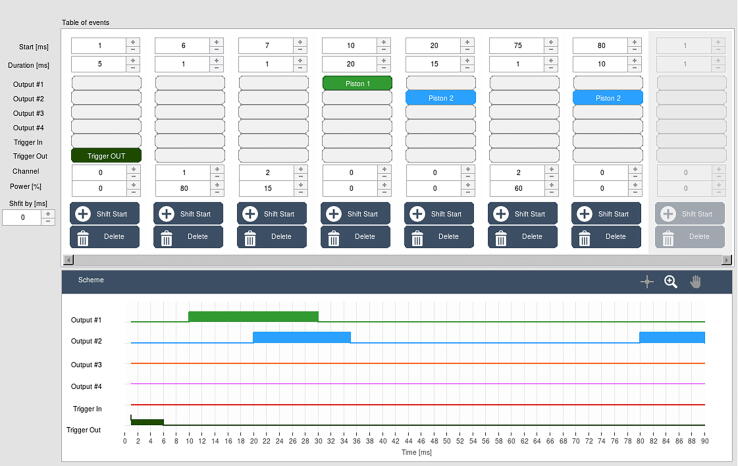
A screenshot from PC software showing the table of events used to test the device operation. This sequence was used to prepare fast-frame movie available for download.

The device is designed as a syringe driver that can be used in devices for investigating the kinetics of chemical reactions on millisecond and second time scales. Accordingly, a corresponding test was performed in which two solutions (250 μl) were mixed by simultaneously injecting them into a third syringe that was stopped immediately after mixing. This experiment was equivalent to the widely used stopped-flow experiment. After incubation for 50 ms, the mixture was ejected from the third syringe by a second syringe driver. The first syringe contained dichlorophenolindophenol, the color of which depends on pH (blue at high pH and red at low pH), and the second syringe contained a hydrochloric acid solution. Change in the color of the solution from blue to red indicated full and rapid mixing of these two solutions. Injekt-F Solo Luer 1-ml syringes were used for the test, and a T-shaped mixer made of plexiglass was used for observing the mixture. The diameter of channels inside the mixer was 1.2 mm. The sequence of the applied pulses is presented in Fig. 8.

**Fig. 9:**
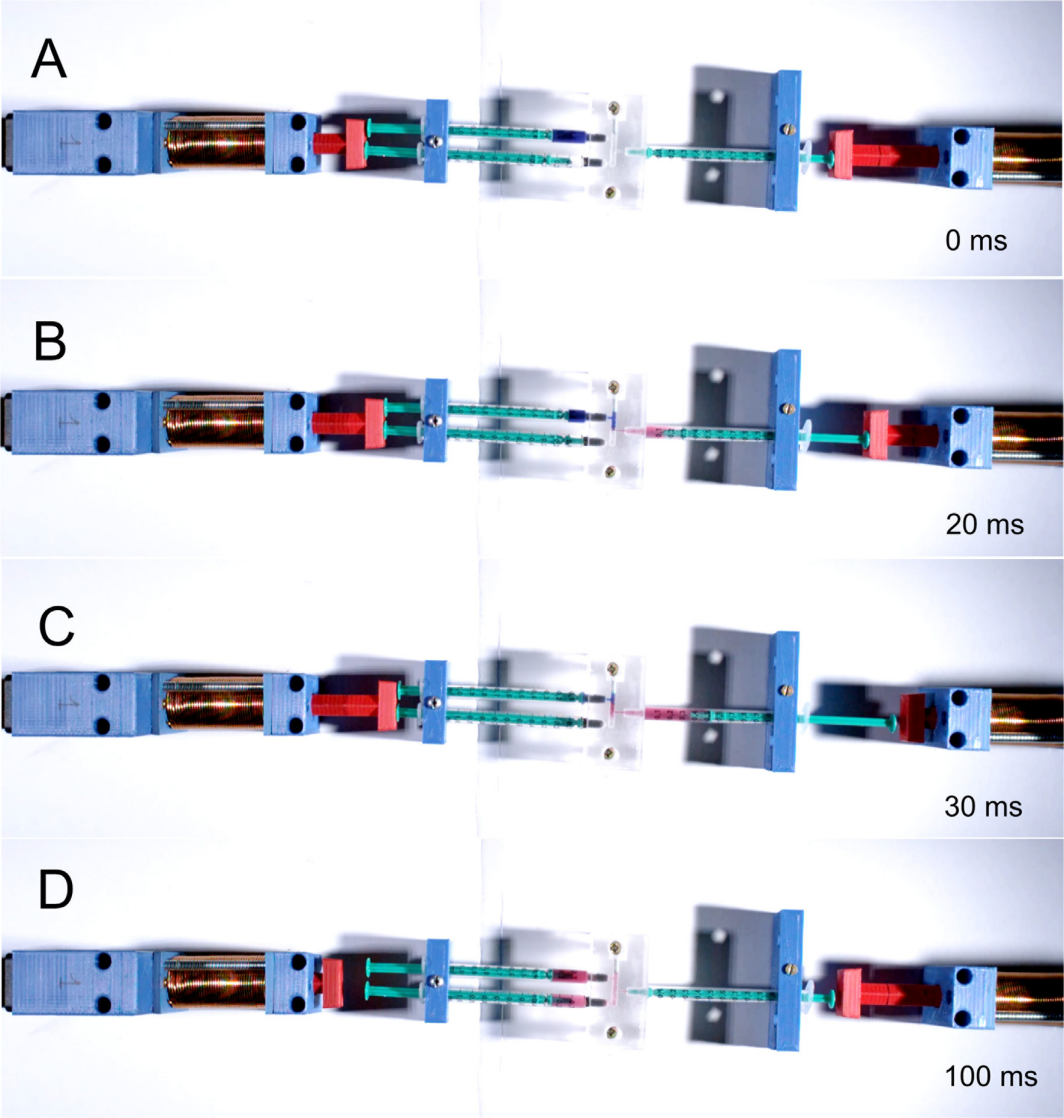
Frames extracted from the fast-frame movie (A–D) at different time points after starting the test sequence programmed as shown in Fig. 8.

The test was recorded using a Phantom Miro LC321S high-speed camera, at a speed of 2000 frames/s. Recording was done in cooperation with EC TEST Systems (Kraków). The video is available for download in the pictures_movie.zip package. The four frames of the movie are shown in Fig. 9. The A–C frames show the mixing of the reagent during the first pulse, and the D frame shows the state at the end of sequence. The device allows rapid injection of solutions from syringes, which is critical for proper mixing of reagents and for investing the kinetics of chemical reactions on millisecond and second time scales.

## Declaration of Competing Interest

The authors declare that they have no known competing financial interests or personal relationships that could have appeared to influence the work reported in this paper.
